# The geography of COVID-19 misinformation: using geospatial maps for targeted messaging to combat misinformation on COVID-19, South Africa

**DOI:** 10.1186/s13104-021-05886-0

**Published:** 2021-12-24

**Authors:** Lucy Chimoyi, Tonderai Mabuto, Tanyaradzwa Dube, Nasiphi Ntombela, Tshegang Nchachi, Dakalo Tshisebe, Candice M. Chetty-Makkan, Geoffrey K. Setswe

**Affiliations:** 1 The Aurum Institute, Johannesburg, South Africa; 2 Health Economics and Research Office, Johannesburg, South Africa; 3 Department of Internal Medicine, School of Clinical Medicine, Faculty of Health Sciences, University of the Witwatersrand, Johannesburg, South Africa; 4 Department of Health Studies, University of South Africa, Pretoria, South Africa; 5 The Aurum Institute, Aurum House, The Ridge, 29 Queens Road, Parktown, Johannesburg, 2193 South Africa

**Keywords:** Targeted interventions, COVID-19, Misinformation, Geospatial, South Africa

## Abstract

**Objective:**

The proliferation of false information on COVID-19 mostly through social media is adversely affecting control efforts. The objective of this study was to identify areas where targeted effective messaging can be useful in demystifying misinformation against COVID-19.

**Results:**

The study showed high levels of misinformation on COVID-19 in the study area [mean score 2.71; standard deviation (SD) 1.5]. The highest levels of misinformation were observed in Dr. Ruth Segomotsi Mompati district, North West province (mean score: 3.84; SD: 2.1) and Sedibeng district, Gauteng province (mean score: 3.56; SD 1.7). Higher levels of misinformation were reported by those aged 18–24 years (mean score: 3.48; SD: 1.8), and men (mean score: 2.73; SD: 1.8). Across the two provinces, we identified geospatial hot and coldspots of misinformation highlighting the need to implement point of care strategies such as targeted messaging. Findings showed the need for targeted interventions to young people, students, those with low levels of education and the self-employed in the two districts more importantly, as South Africa expands its nationwide vaccination roll-out.

**Supplementary Information:**

The online version contains supplementary material available at 10.1186/s13104-021-05886-0.

## Introduction

Misinformation, including myths and misconceptions about COVID-19 transmission and prevention influence the success of public health response efforts to control the epidemic [1]. Importantly, misinformation may result in failure to adopt evidence-informed prevention control measures such as hand hygiene, wearing of protective face masks, social distancing, and more recently—vaccination against COVID-19 [1]. Although information alone does not influence uptake of interventions, several health behaviour frameworks highlight the central role that information plays in influencing attitudes, beliefs and resultant behaviours. Besides the actual COVID-19 disease pandemic, countries are faced with fighting a parallel pandemic, commonly referred to as an ‘infodemic’ of misinformation [2].

Geographic information systems (GIS) and online real- or near-real-time mapping of disease cases across space and time, are becoming indispensable for the timely shaping of an effective response [3]. Application of GIS in guiding COVID-19 response is increasing as it enhances the understanding and control of COVID-19. Interactive web-based maps and dashboards have been used track COVID-19 cases, deaths and recoveries for allocation of resources [3]. The value of maps has broadened our understanding and tracking of infectious diseases through the possibilities for analysing, visualising and detecting patterns of disease [4]. Researchers have employed geospatial techniques to predict the speed and magnitude of transmission, and assess the spatiotemporal dynamics of supply and demand for medical resources to optimize resource allocation [5]. In South Africa, geospatial analysis has been applied in mapping the capability and capacity of health system in response to COVID-19. This identified at a provincial-level, the need to increase intensive care unit bed capacity in response to increasing severe COVID-19 cases [6]. In Cape Town, geospatial analysis showed how difficult it was to achieve social distancing in two high density informal settlements highlighting the need for implementing lockdown at a community rather than household level [7].

As the global response shifts towards biomedical responses to control COVID-19, examining the prevalence of misinformation “by place” is of increasing importance considering that as the epidemic matures—more localised responses will be required for sporadic outbreaks. Focusing on the place is important because there are many unmeasurable factors that make geographical locations unique in terms of socio-economic, demographic, or spatial factors. As countries continue reporting on breakthrough and incident cases, and as they start reporting on vaccine coverage, this information can be superimposed onto the prevalence of misinformation among other variables that influence uptake of interventions. In this paper we present how we utilized secondary data collected from an online survey to geographically visualize the distribution of misinformation on COVID-19 among the South African public as a first step in identifying areas for targeted interventions.

## Main text

### Methods

#### Survey design, sites, population and outcome measurement

We conducted an online cross-sectional survey with participants from Gauteng (high burden) and North West (low burden) provinces, South Africa between May 21, 2020 and June 5, 2020. At the time of this survey, Gauteng province had reported between 2453 and 5215 confirmed cases and was ranked third highest after Western and Eastern Cape compared to other provinces. North West had reported between 77 and 405 confirmed cases and was ranked amongst the lowest.

Participants were from five districts in the Gauteng province (Ekurhuleni, Johannesburg, Tshwane, Sedibeng and West Rand) and four districts in the North West province (Bojanala, Dr. Kenneth Kaunda, Ngaka Modiri Molema and Ruth Segomotsi Mompati). We targeted participants  ≥ 18 years, with access to a mobile device and internet connectivity and ability to complete the questionnaire in English. We administered an online survey questionnaire (Additional file 1) and participants were requested to enable their geographical location to record their geographical positions. We subsequently conducted an exploratory geospatial analysis to identify areas where targeted effective messaging can be useful in demystifying misinformation on COVID-19. We assessed misinformation on COVID-19 by calculating the mean score of responses across six inaccurate statements with “False/True/Unsure” responses. The inaccurate statements were as follows: “Getting a vaccination against flu or pneumonia can help the body fight against the new Coronavirus”, “Gargling mouthwash/salty water can protect a person from getting the new Coronavirus”, “Eating garlic can help the body to fight against the new Coronavirus”, “Taking antibiotics can help the body to fight against the new Coronavirus”, “You can use a hand dryer instead of washing your hands with soap and water to kill any virus that may be on your hands” and “Using traditional herbs to treat the symptoms of coronavirus”. Unsure or true responses were assigned a scoring of 1 for each of the six inaccurate statements whereas those who responded to false, were assigned a scoring of 0. The responses were summed up and a mean score calculated for each participant. Scores  ≥ 2 were considered high scores for misinformation and those  ≤ 1 were low scores. The final analytical dataset contained responses with geographical coordinates and from those that responded to the six statements (see Additional file 2).

#### Data management and analysis

Data was captured in a REDCap database and manipulation and analyses were conducted in STATA 14 software [8]. Individual responses on demographics and sources of COVID-19 information were summarized using frequencies and percentages whereas misinformation was summarized using means and standard deviations (SD). Fischer’s exact tests determined differences in demographic characteristics across districts. We used the kriging function in ArcGIS to calculate an average value for locations with no data using values from nearby weighted locations and identify hotspots or clusters of areas with high levels of misinformation about COVID-19. All maps were produced using ArcMap software v.10.7.1 [9] and its spatial analyst extension.

### Results

#### Demographic characteristics of study population

From 1076 participants, a total of 909 participants mostly from Gauteng province (n  = 661, 72.7%), of black descent (n  = 764, 83.9%), female (n  = 528, 57.8%), not married (n  = 497, 54.7%), employed (n  = 594, 65.1%) and of average age of 36.9 years [standard deviation (SD): 10.8] responded to six inaccurate statements which implied misinformation and recorded their geographical coordinates. Across the districts, the highest proportion of male (n  = 112, 31.8%) and female (n  = 168, 31.8%) participants were from Johannesburg. In Ekurhuleni, most participants were younger participants (18–24 years) (n  = 16, 14.8%). In Johannesburg, most participants who believed misinformation were aged between 25 and 39 years from Johannesburg (n  = 161, 33.5%), those between 40 and 59 years from Tshwane (n  = 88, 30.9%) and those above 60 years from Tshwane (n  = 7, 24.1%) and Dr. Kenneth Kaunda (n  = 7, 24.1%). The demographic characteristics differed significantly across districts and are shown in Additional file 3. Most participants possessed post-secondary school education (n  = 714, 78.5%) where the highest proportion was reported in Johannesburg (n = 200, 28.0%), Gauteng and the lowest in Dr. Ruth Segomotsi Mompati (n  = 6.0%), North West. Most unemployed participants resided in Johannesburg (n  = 178, 30.0%).

#### Sources of COVID-19 information

Most information on COVID-19 was sourced from social media platforms (n  = 749, 69.6%) and television (n  = 815, 75.7%). Additional sources included conversations with family, friends and work colleagues (n  =  507, 47.3%), radio (n  = 450, 41.8%) and healthcare workers (n  = 425, 39.5%). Few received information from magazines and newspapers (n  = 161, 15.0%). Most participants believed in the inaccurate information about COVID-19 [mean score for misinformation was 2.71 (SD: 1.8)] differed across districts (p value  = 0.005) as shown in Table 1. Although misinformation was high in the study area, participants who mostly believed in misinformation about COVID-19 were from Dr. Ruth Segomotsi Mompati (mean score: 3.84; SD: 2.1), North West and Sedibeng (mean score: 3.56; SD: 1.7) District, Gauteng Province. Most young people (18–24 years) (mean score: 3.48; SD: 1.8) and men (mean score: 2.7; SD: 2.73) believed in the inaccurate information on COVID-19.

**Table 1:** Means scores of misinformation on COVID-19 by district

Province	District	Mean (SD)
Gauteng	Johannesburg	2.68 (1.7)
	Tshwane	2.49 (1.8)
	Ekurhuleni	2.81 (1.7)
	West Rand	2.68 (1.5)
	Sedibeng	3.56 (1.7)
North West	Bojanala	2.90 (1.8)
	Dr. Kenneth Kaunda	2.44 (1.5)
	Dr. Ruth Segomotsi Mompati	3.84 (2.1)
	Ngaka Modiri Molema	2.89 (1.9)

#### Hotspots and coldspots of misinformation on COVID-19

**Fig. 1:**
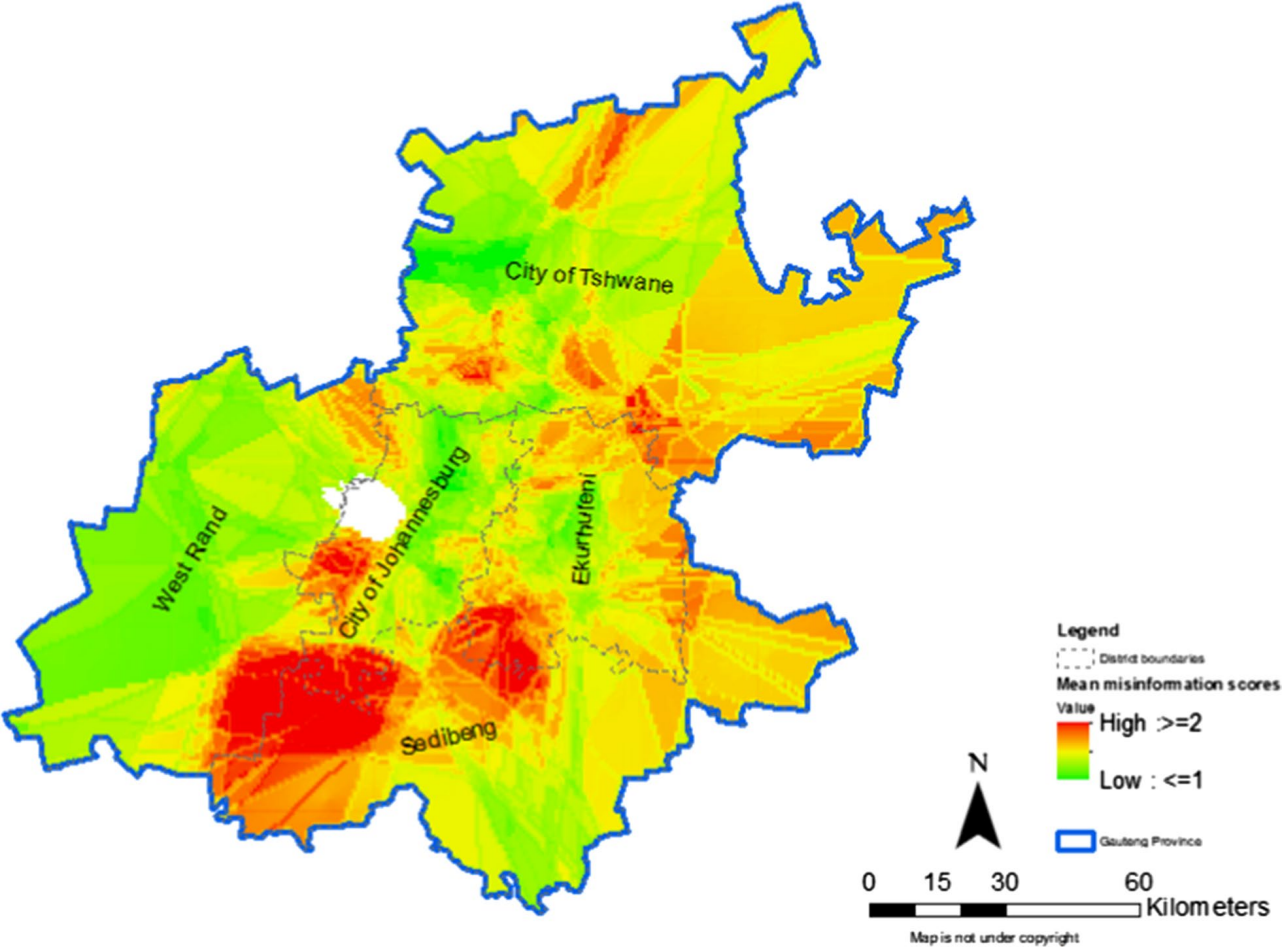
Hotspots and coldspots for misinformation on COVID-19 in Gauteng province

Figure 1 shows the distribution of misinformation on COVID-19 across Gauteng province. The hotspots of misinformation on COVID-19 were observed in Sedibeng towards the northern and north western parts of the district. Hotspots of misinformation on COVID-19 in Ekurhuleni were seen in the south western, far eastern and northern areas. In Johannesburg, these hotspots were observed towards the south western and southernmost areas.

**Fig. 2:**
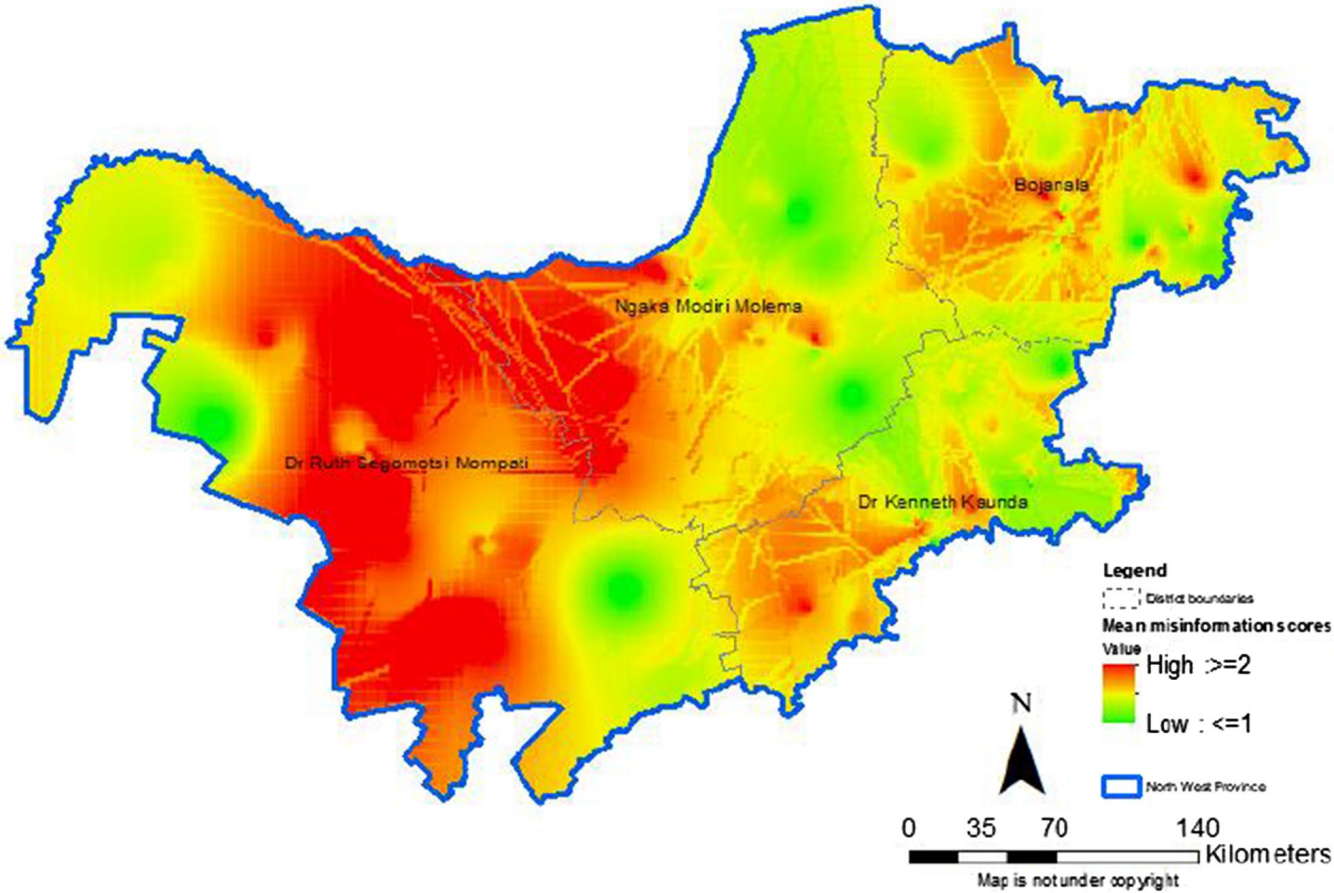
Hotspots and coldspots for misinformation on COVID-19 in North West province

In the North West province, hotspots of misinformation on COVID-19 were observed mainly in Dr. Ruth Segomotsi Mompati District (Fig. 2). Hotspots of misinformation were also observed in Ngaka Modiri Molema District bordering Dr. Ruth Segomotsi Mompati. Coldspots of misinformation were observed in the north western areas of the province.

### Discussion

These findings provide preliminary insights in the distribution of misinformation on COVID-19 in two provinces. Findings show that the level of misinformation on COVID-19 was high among the study population, particularly in younger people and men. In addition, the highest levels of misinformation were observed in Sedibeng and Dr. Ruth Segomotsi Mompati Districts. Maps showed hotspots of misinformation on COVID-19 in areas along boundaries of adjacent districts. Significant differences in demographics were observed across the nine districts. The findings also show heterogeneity of responses on misinformation about COVID-19 which included eating garlic, using mouth wash and traditional herbs to prevent COVID-19.

Similar falsehoods on how to prevent COVID-19 were reported in a study conducted in two developed countries [10]. The need for continuous circulation of accurate information about COVID-19 through social media platforms to dispel these myths is necessary even as the vaccination roll-out program intensifies. This need is greater in this context as Facebook, Twitter and WhatsApp were the preferred sources of information in this survey. Some studies also found misconceptions on how to prevent acquisition of COVID-19, including beliefs in falsehoods circulated through social media [10–14]. An opportunity to correct misconceptions in this platform is necessary. In addition, to social media platforms, television was the most preferred source of information, and this had the capability to reach more members of the public including those with no access to social media platforms. This finding was contrary to a similar online survey conducted in South Africa during lockdown level 5 where majority of respondents relied on government sources for updates and information [14]. Notably, government sources relied on local and international researchers and scientists to ensure factual information was provided. However, since a large proportion from this survey accessed social media platforms, continuous health promotion is required to counter the non-factual information as the measures introduced during lockdown ease. Understanding the distribution of misinformation of COVID-19 amongst the public during any pandemic is necessary to provide targeted evidence-based solutions that inform government policies and other implementing partners.

Our study is among the first to map geospatial hotspots for misinformation on COVID-19 in two provinces in South Africa. These hotspots require point of care strategies to stop the spread of misinformation through social media, particularly as South Africa steps up the vaccination programme. In order to contain COVID-19 in South Africa, modelling studies have shown that a vaccination coverage of 94.4% using a vaccine with an efficacy of 70% is needed [15]. Application of COVID-19 vaccination point of care strategies ensures production of fast actionable results in the control of infectious diseases [16]. Overall, our findings suggest targeting COVID-19 messaging to men and young people. As the vaccination programme rolls-out in South Africa, concerted efforts to combat vaccine hesitancy and subsequently increase uptake, will be required in Dr. Ruth Segomotsi Mompati and Sedibeng Districts. Appropriate messaging should be directed to students, young people (18–24 years), those of low education level and self-employed in these two districts. A systematic review highlighted the role of social media in spreading accurate information and misinformation about COVID-19 across all age-groups [2]. Therefore, implementers’ nationwide need to increase their presence of COVD-19-related activities on social media as has been done through television. Collaboration with public health providers within and between adjacent districts is encouraged to target the hotspots observed in areas sharing district boundaries.

### Limitations

The present findings should be interpreted with caution owing to several limitations.

Firstly, since the participants were from two provinces, they might not fully reflect the entire South African population, which limits the generalization of the findings. However, the findings may be representative of the situation in high and low burden settings. Secondly, there may be self-report response bias from self-collected data in this survey. This likely introduced variation that was adjusted during geospatial analysis. Despite the limitations, findings from this survey adds to the growing body of literature on misinformation on COVID-19 which may improve implementation of COVID-19 control efforts including the on-going vaccination roll-out.

## Supplementary Information

**Additional file 1: Questionnaire S1. **Online questionnaire.

**Additional file 2: Table S1.** Dataset.

**Additional file 3: Table S2.** Table showing demographic characteristics across districts.

## Data Availability

All data generated or analysed during this study are included in this published article (and its Additional files).

## Abbreviations

COVID-19: Coronavirus disease
GIS: Geographical information systems
SD: Standard deviation
