# Supplementary material for: The geography of COVID-19 misinformation: using geospatial maps for targeted messaging to combat misinformation on COVID-19, South Africa
Source: BMC Res Notes. 2021 Dec 24;14:468. doi: 10.1186/s13104-021-05886-0 (PMC8708513; doi:10.1186/s13104-021-05886-0)
Supplement: Supplementary file 1 — Additional file 1: Questionnaire S1. Online questionnaire. [file 13104_2021_5886_MOESM1_ESM.pdf]

THE AURUM INSTITUTE

KAB COVID19 Survey: Assessing the Knowledge, Attitudes and Behaviours (KAB) of South Africans about the Novel Corona virus (COVID-19) among the South African Public during National Lockdown

KAB Questionnaire COVID-19 Lockdown

Thank you for agreeing to participate in our research and taking the time to answer our questions. We are interested in knowing about people's knowledge, attitudes, perceptions and behaviours related to COVID-19. Some of these questions are sensitive, but it is very important that we know the truth about the behaviours happening in your community. There are no right or wrong answers. We are still trying to understand the Coronavirus, so much of the time there is no correct answer. Please just give it your best guess. All you need to do is to fill out the answers to the best of your knowledge. Please be honest when answering these questions. We will keep your answers confidential. Confidential means we will not tell your answers to anyone outside the research group. Before you start with the questionnaire, please turn off your e-mail, and music, so that you can focus on this study. We would like to know what you understand and think about COVID-19. Please give us your honest opinion. Avoid using pamphlets or internet searches to assist you, as we would like to know your personal attitude toward COVID-19. Thank you!

Participant identity number: ________________________________

Time questionnaire started (hh:mm): _________________________

1. **What do you know about Coronavirus and the spread of COVID-19?**

1.1	Have you ever heard about the new Coronavirus or COVID-19	1= Yes<br>0 = No	
1.2	How do you get most of your information about the new Coronavirus?<br>**Can choose multiple options**	1= Social media (like WhatsApp Facebook, Twitter, etc.)<br>2= Searching internet for information (Google)<br>3= Television<br>4= Radio<br>5= Magazines and newspapers<br>6= Conversation with other people (e.g. family, friends, work colleagues)<br>7= Healthcare workers<br>8= Other	
1.2.1	**If code 9 above**	Describe here→	
1.3	What would you do if you develop a fever or cough and suspect that you might have been exposed to or could be possibly infected with Coronavirus?	1= Take public transport to a clinic or hospital<br>2= Ask someone to drive me to a clinic or hospital<br>3= Stay home and call my doctor<br>4= Take public transport to go to a pharmacy to buy medication for cough and fever<br>5 = Ask someone to drive me to a pharmacy to buy medication for cough and fever<br>6= Call the toll free COVID-19 emergency number<br>7 = Stay at home and just wait for the cough and fever to go away	
1.4	Are those with other health problems more likely to die from an infection with	0= No<br>1= Yes<br>2= Unsure	

	the new coronavirus disease than those without any other health problems?		

2. **Transmission of the new Coronavirus**

**Please indicate whether you believe these statements to be true or false**			
2.1	The Coronavirus is mainly spread from person-to-person when an infected person sneezes or coughs and droplets of their saliva land in the mouth or nose of a person who is nearby	0= False<br>1= True<br>2= Unsure	
2.2	Only older adults can become infected with the new coronavirus	0= False<br>1= True<br>2= Unsure	
2.3	Only people who have travelled outside of South Africa can become infected with the new Coronavirus	0= False<br>1= True<br>2= Unsure	
2.4	Most people who are infected from the new Coronavirus die from it	0= False<br>1= True<br>2= Unsure	
2.5	The new Coronavirus can be spread by touching a surface or object that has the virus on it and then touching your own mouth, nose, or possibly your eyes	0= False<br>1= True<br>2= Unsure	

3. **Preventing transmission**

**Please indicate whether you believe these statements to be true or false about action to prevent being infected by the Coronavirus**			
3.1	Wearing a face mask is the most important way to prevent the spread of the new Coronavirus in South Africa	0= False<br>1= True<br>2= Unsure	
3.2	Getting a vaccination against flu or pneumonia can help the body fight against the new Coronavirus	0= False<br>1= True<br>2= Unsure	
3.3	Gargling mouthwash/salty water can protect a person from getting the new Coronavirus	0= False<br>1= True<br>2= Unsure	
3.4	Washing hands regularly with soap and water helps to kill any viruses that may be on the hands	0= False<br>1= True<br>2= Unsure	
3.5	Eating garlic can help the body to fight against the new Coronavirus	0= False<br>1= True<br>2= Unsure	
3.6	Avoiding close contact with people who are sick, can reduce the chances of becoming infected with the new Coronavirus	0= False<br>1= True<br>2= Unsure	

3.7	Taking antibiotics can help the body to fight against the new Coronavirus	0= False<br>1= True<br>2= Unsure	
3.8	You can use a hand dryer instead of washing your hands with soap and water to kill any virus that may be on your hands	0= False<br>1= True<br>2= Unsure	
3.9	Putting sesame oil on your skin or under the nose can block the corona virus from entering the body	0= False<br>1= True<br>2= Unsure	
3.10	Avoiding touching the eyes, nose and mouth with unwashed hands can reduce the chances of becoming infected with the new Coronavirus	0= False<br>1= True<br>2= Unsure	
3.11	Regularly rinsing your nose with saline can reduce the chances of a person becoming infected with the new Coronavirus	0= False<br>1= True<br>2= Unsure	
3.12	Wiping the handles of trolleys or baskets in grocery stores with alcohol wipes can reduce the chances of getting the new Coronavirus	0= False<br>1= True<br>2= Unsure	
3.13	Wearing gloves is more effective than regular washing hands for the majority of South Africans to reduce the spread of the new Coronavirus in South Africa	0= False<br>1= True<br>2= Unsure	

4. **Common signs or symptoms of an infection with the new Coronavirus?**

**Please indicate whether you believe these statements to be true or false about the common signs and symptoms of an infection with Coronavirus**			
4.1	Nose bleeds	0= False<br>1= True<br>2= Unsure	
4.2	Cough	0= False<br>1= True<br>2= Unsure	
4.3	Fever	0= False<br>1= True<br>2= Unsure	
4.4	Skin rash	0= False<br>1= True<br>2= Unsure	
4.5	Constipation	0= False<br>1= True<br>2= Unsure	
4.6	Shortness of breath	0= False<br>1= True<br>2= Unsure	
4.7	Frequent urination	0= False<br>1= True<br>2= Unsure	

5. **Perceived threats of the new Coronavirus?**

What level of threat do you think the Coronavirus poses to each of the following:			
5.1	You and your family	0= High threat<br>1= Moderate threat<br>2= Low threat<br>3 = Don't know	
5.2	Jobs for of people in South Africa	0= High threat<br>1= Moderate threat<br>2= Low threat<br>3 = Don't know	
5.3	People who are elderly	0= High threat<br>1= Moderate threat<br>2= Low threat<br>3 = Don't know	

6. **What do you think about the actions and steps that the government put in place to manage spread of the Coronavirus? (please write in the space provided)**

6.1	What do/did you think about the lockdown?	
6.2	What are/were you most afraid of losing during the lock down?	
6.3	What do/did you most value during the lockdown?	
6.4	Why do you think some people did not follow the guidance by government on the lockdown?	
6.5	How has/did your behaviour change during the lockdown?	

7. **Have you done any of the following in the last seven days to keep yourselves safe from catching an infection with the new coronavirus during the lockdown?**

7.1	Wearing a face mask	0= No<br>1= Yes<br>2= Not applicable	
7.2	Often gargling with mouthwash to protect yourself from infection	0= No<br>1= Yes<br>2= Not applicable	
7.3	Washing your hands or using hand sanitizer more frequently	0= No<br>1= Yes<br>2= Not applicable	
7.4	Eating garlic to help your body fight any infection from the new Coronavirus	0= No<br>1= Yes<br>2= Not applicable	
7.5	Avoiding close contact with people who are sick	0= No<br>1= Yes<br>2= Not applicable	
7.6	Taking antibiotics	0= No	

		1= Yes<br>2= Not applicable	
7.7	Using a hand dryer to kill any virus on hands	0= No<br>1= Yes<br>2= Not applicable	
7.8	Putting sesame oil on your skin or nose to prevent virus entering your body	0= No<br>1= Yes<br>2= Not applicable	
7.9	Avoiding touching your eyes, nose and mouth with unwashed hands	0= No<br>1= Yes<br>2= Not applicable	
7.10	Regularly rinsing your nose with saline to prevent infection with new Coronavirus	0= No<br>1= Yes<br>2= Not applicable	
7.11	Using wipes to disinfect trolleys and shopping baskets	0= No<br>1= Yes<br>2= Not applicable	
7.12	Stocking up on groceries or essential items	0= No<br>1= Yes<br>2= Not applicable	
7.13	Praying	0= No<br>1= Yes<br>2= Not applicable	
7.14	Avoiding using public transport	0= No<br>1= Yes<br>2= Not applicable	

8. **Please rate your opinion about the following statements?**

		Strongly Agree (1)	Moderately Agree(2)	Agree (3)	Disagree (4)	Moderately Disagree(5)	Strongly Disagree(6)
8.1	The measures put in place by the South African government are sufficient to control the spread of the new Coronavirus						
8.2	The media is exaggerating the seriousness of the virus						
8.3	I know how to protect myself and family from the new Coronavirus if transmission is widespread in South Africa						

9. **Socio-demographic characteristics**

**Can you please tell us more about you:**			
9.1	**To which gender do you most identify?**	0= Man<br>1= Woman<br>2= Transgender<br>3 = Additional gender category/identity (please specify)<br>4 = Prefer not to disclose	
9.2	**How old are you?**	Code in years	
9.3	**What is your date of birth?**	Write in date	___ ___/ ___ ___ ___/ ___ ___ ___ ___<br>DAY / M O N / Y Y Y Y
9.4	**How would you describe yourself in terms of population group? Please pick <u>one</u> from these options.**	1= Black African<br>2= Coloured<br>3= Indian or Asian<br>4= White<br>5= Other	
9.4.1	**If code 5 above**	If code 5 above specify here→	
9.5	**What is the highest level of education that you have completed?<br>Please pick <u>one</u> from these options.**	1= Never attended school or never completed any level of school<br>2= Completed primary school (Grades 1 – 8 or Standard 1 – 7)<br>3= Completed Secondary School (Grades 10 -12 or Standard 6 – 10)<br>4= Completed a post-matric qualification (e.g. higher certificate, diploma or degree)<br>5= Other (specify)	
9.5.1	**If code 10 above**	Write other education here→	
9.6	**In which province do you currently reside?**	1= Gauteng<br>2= North West<br>3= Other	
9.6.1	**If code 3 above**	Write other province→	
9.7	**How would you describe your employment situation?<br>Please pick <u>one</u> from these options.**	1= Unemployed looking for work<br>2= Unemployed not looking for work<br>3= Unable to work - Receive social grant<br>4= Student/pupil/learner	

		5= Self-employed – part time less than 40 hours per week<br>6= Self-employed – full time 40 hours or more per week<br>7= Employed, less than 40 hours per week<br>8= Employed, full time (40 hours or more)<br>9= Other	
9.7.1	**If code 9 above**	Write employment situation→	
9.8	**What has been your main source of income in the last 12 months? Please pick <u>one</u> from these options.**	1= Employment (full-time, part-time, self-employment)<br>2= Pension<br>3= Social grants (including unemployment insurance fund (UIF))<br>4= Investments (including rentals)<br>5= Remittances or gifts from family<br>6= No source of income<br>7= Other	
9.8.1	**If code 7 above**	Write primary source of income here→	
9.9	What is the income category that best describes your gross monthly income before deductions and including all sources of income?	1= Less than R5000<br>2= R5000 – R10,000<br>3= R10,000 - R19,999<br>4= R20,000 - R29,999<br>5= R30,000 - R39,999<br>6= R40,000 - R49,999<br>7= R50,000 - R59,999<br>8= R60,000 - R69,999<br>9= R70,000 - R79,999<br>10= R80,000 - R89,999<br>11= R90,000 - R99,999<br>12= R100,000 - R149,999<br>13= R150,000 or more<br>14= Don't know<br>15= None or not applicable	
9.10	What is your marital status?	1= Married or living with someone as married<br>2= Never married<br>3= Widowed<br>4= Divorced/separated<br>5= Other	
9.10.1	If code 5 above	Write marital status here→	

Time questionnaire ended (hh:mm): ___________________________
