# Supplementary material for: The geography of COVID-19 misinformation: using geospatial maps for targeted messaging to combat misinformation on COVID-19, South Africa
Source: BMC Res Notes. 2021 Dec 24;14:468. doi: 10.1186/s13104-021-05886-0 (PMC8708513; doi:10.1186/s13104-021-05886-0)
Supplement: Supplementary file 3 — Additional file 3: Table S2. Table showing demographic characteristics across districts. [file 13104_2021_5886_MOESM3_ESM.docx]

*Table S2: Demographic characteristics of participants by districts*

| Characteristic | | Gauteng province (High burden)  (N= 661, 72.7%) | | | | | North West province (Low burden)  (N= 248, 27.3%) | | | | p-value |
| --- | --- | --- | --- | --- | --- | --- | --- | --- | --- | --- | --- |
|  |  | Johannesburg  (n=289, 31.7%) | Tshwane  (n=199, 21.8%) | Ekurhuleni  (n=138, 15.1%) | West Rand  (n=19, 2.1%) | Sedibeng  (n=27, 3.0%) | Bojanala  (n=77, 8.43%) | Dr Kenneth Kaunda  (n=100, 11.0%) | Dr Ruth Mompati  (n=19, 2.1%) | Ngaka Modiri Molema  (n=33, 6.3%) |  |
| Gender | Male  (n=352, 38.6%) | 112 (31.8%) | 98 (27.8%) | 47 (13.4%) | 6 (1.7%) | 13 (3.7%) | 26 (7.4%) | 37 (10.5%) | 3 (0.9%) | 10 (2.8%) | 0.001 |
|  | Female  (n=528, 57.8%) | 168 (31.8%) | 98 (18.6%) | 84 (15.9%) | 13 (2.5%) | 13 (2.5%) | 47 (8.9%) | 60 (11.4%) | 12 (2.3%) | 33 (6.3%) |  |
| Age categories (years) | 18-24  (n=108, 12.0%) | 38 (5.2%) | 9 (8.3%) | 16 (14.8%) | 0 (0.0%) | 4 (3.7%) | 9 (8.3%) | 11 (10.2%) | 10 (9.3%) | 11 (10.2%) | <0.001 |
|  | 25-39  (n=480, 53.2%) | 161 (33.5%) | 93 (19.4%) | 81 (16.9%) | 9 (1.9%) | 14 (2.9%) | 45 (9.4%) | 50 (10.4%) | 6 (1.3%) | 21 (4.4%) |  |
|  | 40-59  (n=285, 31.6%) | 82 (28.8%) | 88 (30.9%) | 36 (12.6%) | 9 (3.2%) | 7 (2.5%) | 20 (4.0%) | 31 (10.9%) | 1 (3.5%) | 11 (3.9%) |  |
|  | 60+  (n=29, 3.2%) | 4 (13.8%) | 7 (24.1%) | 3 (10.3%) | 1 (3.5%) | 2 (6.9%) | 3 (10.3%) | 7 (24.1%) | 1 (3.5%) | 1 (3.5%) |  |
| Race | Black  (n=764, 83.9%) | 246 (32.2%) | 175 (22.9%) | 117 (15.3%) | 15 (2.0%) | 24 (3.1%) | 71 (9.3%) | 56 (7.3%) | 18 (2.4%) | 42 (5.5%) | <0.001 |
|  | Mixed  (n=22, 2.4%) | 7 (31.8%) | 4 (18.2%) | 2 (9.1%) | 0 (0.0%) | 1 (4.6%) | 2 (9.1%) | 5 (22.7%) | 0 (0.0%) | 1 (4.6%) |  |
|  | Indian/Asian (n=21, 2.3%) | 13 (61.9%) | 2 (9.5%) | 5 (23.8%) | 0 (0.0%) | 0 (0.0) | 1 (4.8%) | 0 (0.0%) | 0 (0.0%) | 0 (0.0%) |  |
|  | White  (n=100, 11%) | 22 (22.0%) | 16 (16.0%) | 14 (14.0%) | 4 (4.0%) | 2 (2.0%) | 3 (3.0%) | 37 (37.0%) | 1 (1.0%) | 1 (0.0%) |  |
| Education level | Primary  (n=17, 1.9%) | 9 (52.9%) | 1 (5.9%) | 0 (0.0%) | 0 (0.0%) | 5 (29.4%) | 0 (0.0%) | 0 (0.0%) | 2 (11.8%) | 0 (0.0%) | <0.001 |
|  | Secondary  (n=177, 19.5%) | 80 (45.2%) | 23 (13.0%) | 26 (14.7%) | 2 (1.1%) | 7 (4.0%) | 15 (8.5%) | 13 (7.3%) | 5 (2.8%) | 6 (3.4%) |  |
|  | Post-secondary (n=714, 78.5%) | 200 (28.0%) | 174 (24.4%) | 110 (15.4%) | 17 (2.4%) | 15 (2.1%) | 62 (8.7%) | 86 (12.0%) | 12 (1.7%) | 38 (5.3%) |  |
| Employment status | Unemployed (n=199, 21.8%) | 67 (33.7%) | 36 (18.1%) | 25 (12.6%) | 2 (1.0%) | 5 (2.5%) | 21 (10.6%) | 24 (12.1%) | 6 (3.0%) | 13 (6.5%) | <0.001 |
|  | Student  (n=49, 5.4%) | 18 (36.7%) | 4 (8.2%) | 6 (12.2%) | 0 (0.0%) | 2 (4.1%) | 3 (6.1%) | 2 (4.1%) | 6 (12.2%) | 8 (16.3%) |  |
|  | Self-employed (n=66, 7.2%) | 26 (39.4%) | 14 (21.2%) | 5 (7.6%) | 3 (4.6%) | 7 (10.6%) | 4 (6.1%) | 6 (9.1%) | 0 (0.0%) | 1 (1.5%) |  |
|  | Employed  (n=594, 65.1%) | 178 (30.0%) | 142 (23.9%) | 101 (17.0%) | 14 (2.4%) | 13 (2.2%) | 49 (8.3%) | 68 (11.5%) | 6 (1.0%) | 23 (3.9%) |  |
| Currently married | Yes  (n=441, 45.3%) | 119 (29.0%) | 111 (27.0%) | 59 (14.4%) | 9 (2.2%) | 10 (2.4%) | 29 (7.1%) | 60 (14.6%) | 0 (0.0%) | 14 (3.4%) | <0.001 |
|  | No  (n=497, 54.7%) | 170 (34.2%) | 86 (17.3%) | 79 (15.9%) | 10 (2.01) | 17 (3.4%) | 47 (9.5%) | 39 (7.9%) | 18 (3.6%) | 31 (6.2%) |  |

Row percentages shown per district; overall percentage is shown as column percentages
